# Geometric Defects and Icosahedral Viruses

**DOI:** 10.3390/v10010025

**Published:** 2018-01-04

**Authors:** Joseph Che-Yen Wang, Suchetana Mukhopadhyay, Adam Zlotnick

**Affiliations:** 1 Indiana University Electron Microscopy Center, Indiana University, Bloomington, IN 47405, USA; 2 Department of Biology, Indiana University, Bloomington, IN 47405, USA; 3 Molecular and Cellular Biochemistry Department, Indiana University, Bloomington, IN 47405, USA

**Keywords:** capsid, nucleocapsid, hepadnavirus, alphavirus, self-assembly, cryo-electron microscopy

## Abstract

We propose that viruses with geometric defects are not necessarily flawed viruses. A geometric defect may be a reactive site. Defects may facilitate assembly, dissociation, or accessibility of cellular proteins to virion components. In single molecule studies of hepadnavirus assembly, defects and overgrowth are common features. Icosahedral alphaviruses and flaviviruses, among others, have capsids with geometric defects. Similarly, immature retroviruses, which are non-icosahedral, have numerous “errors”. In many viruses, asymmetric exposure of interior features allows for regulated genome release or supports intracellular trafficking. In these viruses, the defects likely serve a biological function. Commonly used approaches for spherical virus structure determination use symmetry averaging, which obscures defects. We suggest that there are three classes of asymmetry: regular asymmetry as might be found in a tailed phage, irregular asymmetry as found, for example, in defects randomly trapped during assembly, and dynamic asymmetry due to Brownian dynamics of virus capsids. Awareness of their presence and recent advances in electron microscopy will allow unprecedented investigation of capsid irregularities to investigate their biological relevance.

## 1. Symmetry and Structure: A Double-Edged Sword

Viruses come in a variety of shapes and sizes: rod-like, spherical, elliptical, and pleomorphic. From a virus’ perspective, the most efficient way to enclose the large volume need for the viral genome is to make a large capsid from many copies of relatively small protein [1]. From an assembly perspective, the arrangement of these proteins is critical and tied to function. When Caspar published X-ray precession photographs of Tomato Bushy Stunt Virus more than 60 years ago, he provided the first experimental evidence of icosahedral symmetry in a spherical virus [2].

Viral symmetry has also played a critical role in structure determination. The realization that non-crystallographic icosahedral symmetry could be utilized to improve phases made the determination of virus crystal structures possible [3], a turning point in structural biology. Symmetry averaging is almost always used in cryo-electron microscopy (cryo-EM) structure determination of icosahedral viruses and capsids. An advantage and disadvantage of electron microscopy is the ability of the experimentalist to discard irregular particles that would perturb averaging and keep only the “shiny” particles. Thus, particles that do not conform with the experimentalists’ expectations might be excluded from the data based on their poor statistical matching to projections of the structure in progress (e.g., [4]) or at the level of 2D or 3D class-averaging [5]. Class averaging sorts images based on their dominant features; averaging of the classified particles allows visualization of details that would otherwise be lost in the noise of a cryo-electron micrograph of a single particle. By retaining only the most uniform particles for a reconstruction and imposing icosahedral symmetry, it is possible to achieve atomic resolution with ≥10^4^ particles. But selection and averaging come at a cost.

Symmetry averaging leads to a loss of asymmetric detail. Some features, such as the organization of the virus genome, though found in every particle, are expected to be obscured by averaging [6]. However, averaging can lead to oversimplification of the virion structure. Partial occupancy or multiple orientations, as with viral scaffold proteins and bound antibodies, can lead to weak, blurred, and confusing density maps. Cryo-EM image reconstructions of Fabs bound to Cowpea Mosaic Virus show unambiguous rod-like protrusions while Fabs bound to hepatitis B virus (HBV) capsids appear to form an overlapping network as an artifact of averaging [7,8]. Similarly, phage P22 scaffold proteins are all but invisible in most reconstructions [9,10] and the karyopherin Importin β forms a delocalized cloud when bound to the flexible C-terminal domain of HBV [11]; these examples may result from the dynamics of the ligand as well as irregular positioning. The herpesvirus portal structure, replacing one icosahedral fivefold vertex, had been obscured by icosahedral averaging [12,13,14]. These examples show how symmetry averaging can disguise or altogether hide features. Defects in the capsid are a special example of an irregular feature.

An icosahedral virus is by definition symmetric. Asymmetry can be imposed by several mechanisms that we term: regular asymmetry, irregular asymmetry, and dynamic asymmetry. In regular asymmetry, particles have a well-defined modification to their symmetry; examples are the preferred attachment of the transferrin receptor to canine parvovirus [15], the polymerases of HBV and cytoplasmic polyhedrosis virus, a reovirus [16,17], and the position of the receptor and genomic organization in phage MS2 [18,19]. Irregular asymmetry is stochastic in nature and is exemplified by defects trapped during capsid assembly as seen in HBV and Ross River Virus (RRV) [20,21]; we argue that these symmetry defects are not indicative of a defective virus. Irregular asymmetry may result in a diverse ensemble of local structures unevenly distributed across a population of viruses; it will be very difficult to reconstruct. Dynamic asymmetry arises because virus particles are “kicking and screaming ‘stochastic’ molecules”. Relatively slow dynamics have been shown biochemically by the exposure of internal components to the exterior of the capsid [22,23]. Faster dynamics have been investigated by whole capsids molecular dynamics, where no two subunits appear exactly the same [24]. In this review, we emphasize the implications of irregular asymmetry.

**Figure 1:**
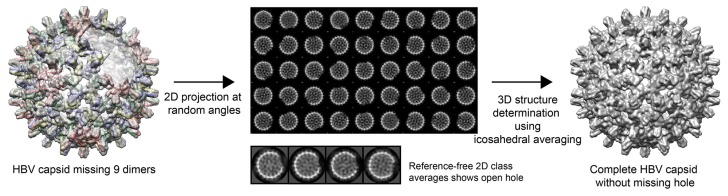
Asymmetric features are eliminated by icosahedral averaging. An electron density map of an hepatitis B virus (HBV) capsid that lacked a cluster of nine dimers around a quasi-sixfold axis was created and low-pass-filtered to 10 Å (**left**). The model was used to computationally produce 1000 particle images at random angles (**center**, top panel). Each particle had a clear nick at its surface. Unsupervised 2D classification clearly showed missing density in each particle (**center**, lower panel). However, when particles were subjected to 3D structure determination with imposed symmetry arising from icosahedral averaging, the missing density was generated and a complete capsid was obtained (**right**).

To demonstrate how averaging can act as a means of generating structure while losing information about symmetry defects, we built an incomplete virus capsid, generated a series of projections, and then calculated an averaged structure (Figure 1). In 2D class averages of the projections, we observed evidence of the defect, but these defects were washed away in an icosahedrally averaged 3D structure. Symmetry averaging completely obscured the presence of an asymmetric gap in the capsid. However, without symmetry averaging, we would not have been able to achieve maximal resolution in the 3D structure.

## 2. Evolutionary Advantages and Limitations of Icosahedral Symmetry

Symmetry contributes to capsid stability. Capsids must balance between a stable entity that protects its genomic material and a labile shell that disassembles at a precise time and cellular location to initiate an infection. When every subunit in the capsid interacts with its full complement of neighbors, the capsid is at an energy minimum. Icosahedral symmetry represents a realization of that energy minimum [25]. The uniform interactions between subunits form an inherent barrier to dissociation, which can result in metastable capsids that persist for months under conditions where they would never assemble; particles with defects may not have this advantage [26,27]. In general, to allow assembly of regular particles, virus capsid proteins make weak intersubunit interactions that allow defects to melt out, while correct interactions between multivalent subunits support further assembly [28]. Conversely, strong interactions can lead to kinetic traps that lock defects into place. To avoid traps, bacteriophages with very stable capsids, such as P22 and HK97, initially assemble as fragile provirions; these later mature into extremely stable particles [29,30]. Thus, the advantages of icosahedral symmetry come with costs – subunit geometry must be ideal, subunit interaction energies have a relatively narrow window, and the resulting capsids’ resistance to dissociation makes them peculiarly unresponsive to its environment.

It is possible to make a spherical capsid without symmetry. Computational studies show that when the number of subunits is relatively small, e.g., 12 subunits, there is a substantial energetic difference favoring the symmetrical 12-mer over incomplete/over-complete complexes; however, for larger oligomers, e.g., > 40-mers, there is a negligible difference between similarly sized complexes that do and do not satisfy icosahedral symmetry [31,32]. Particles with defects can lead to unique local energy minima [33]. A gap or a disclination in a structure can also provide a starting point for dissociation.

## 3. Symmetry Defects Are Not Rare

Viruses with geometric defects are not necessarily defective or “flawed” particles. We define defective particles as ones that are generally not infectious, possibly a result of misassembly or damage during purification. Conversely, virions with geometric defects may be the infectious particles.

Geometric defects can contribute to, and maybe required for, biological function. Hypothetically, a defect may be a scar from nucleation, an effect of the unique features of the genome, or a site for binding to a receptor or initiating dissociation. Below we give some examples from the literature of particles with defects and how the lack of an icosahedral structure may be biologically advantageous.

Retroviruses, particularly human immunodeficiency virus (HIV), are a well characterized, biological example of irregularity. Tomographic structures of immature HIV reveal a partial shell with numerous holes in the Gag protein lattice [34]. Such an incomplete particle presumably requires relatively strong intersubunit contacts for self-assembly and to persist as a stable complex. These defective lattices suggest a kinetic trap, a contrast to an icosahedral capsid. The geometric defects in retroviruses may provide an opening for the maturation protease to cleave Gag into its component proteins including the capsid protein (CA) domain [35]. Of note, in mature HIV (and other retroviruses) CA protein forms conical oligomers, many of which have a seam, suggesting a disclination [36]. Defects such as the seam may play a role in disassembly.

**Figure 2:**
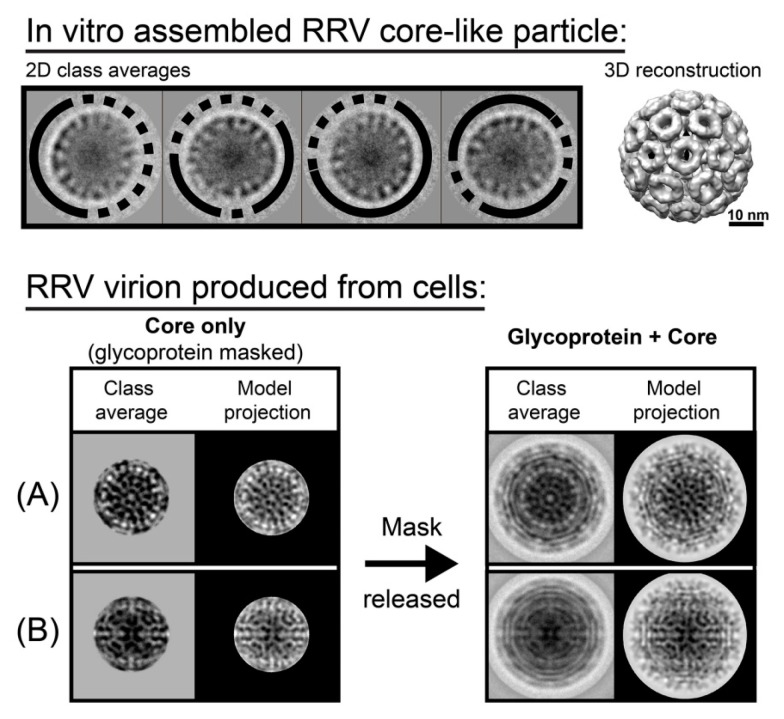
Geometric defects seen in the alphavirus capsid. (**Top**) Reference-free class averages of in vitro assembled Ross River Virus (RRV) core-like particles show substantial density irregularities (dashed arcs) and well-defined areas (solid arcs) around the capsid periphery (upper panel, left). This indicates that particles had large, partially disordered regions, while the other regions were structurally ordered. However, with imposition of icosahedral averaging, these data could be used to generate 3D reconstruction that show a complete ordered structure (upper panel, right). (**Bottom**) Virions had similar results. When aligning virions using only the core region, the glycoprotein region was masked, the class averaged cores were matched with model projections (**A**,**B**, **left panel**, “core only” panels). However, when the mask was removed, it was evident that around 40% of virions had capsid flaws or had cores that were misaligned with the glycoprotein layer (lower panel), suggesting heterogeneity of the core that is inconsistent with icosahedral symmetry. (**A**,**B**, **right panel** “glycoprotein + core” panels) (**A**) An example of good match of both core and glycoprotein layers. (**B**) An example of a mismatch. The core region is consistent with a projection of a model, but the glycoprotein layer has smeared density and poor agreement with the projection, suggestive of circular averaging. (This figure is based on data from [21] and used with permission).

Unlike pleomorphic HIV, alphaviruses show T = 4 icosahedral symmetry in 3D image reconstructions. Alphaviruses are enveloped viruses where the capsid and envelope proteins both appear to be well ordered. However, recent work indicates that, in vitro and in vivo, a substantial fraction of the alphavirus RRV has geometric defects in its capsids (Figure 2 [21]). In vitro assembled capsids appeared uniform by EM and were sufficient for image reconstruction of a T = 4 capsid. However, biochemical studies showed that in vitro assembled cores and cores from authentic alphavirus virions were extremely sensitive to solution conditions [37]. In vitro particles were re-examined by 2D class averaging and every class was found to have substantial defects. Like HIV, there are strong interactions between subunits, and strong association energy may bias assembly to kinetically trapping geometric defects. To determine if incomplete cores were present in mature virions, class averages of RRV virions were performed and capsids in at least 40% of the virions showed evidence of disorder [21]. This is probably a low estimate, as well-ordered envelope influences efforts to classify images. It is hypothesized that defects in the capsid provide a replicative advantage to the virus. During infection, RRV enters the cell via endocytosis; fusion of the envelope with the endosomal membrane releases the capsid into the cytoplasm where it must rapidly fall apart to release viral RNA for translation. The envelope provides a basis for persistence and after its loss; we suggest that defects enable capsid dissociation.

In retrospect, it is not surprising that an incomplete lattice can be used to generate an image reconstruction of a complete icosahedron (e.g., Figure 1). A lattice that is grossly irregular with respect to icosahedral symmetry would be expected to yield blurred density or an absence of density. In most flaviruses, e.g., Dengue and West Nile, reconstructions show a well-ordered envelope and no density for the capsid [38,39,40]. In the recent Zika virus structures, which achieved at least 3.8 Å resolution for the envelope proteins, resolving many side chains, there was no visible density for the capsid layer [41,42]. Though it is not icosahedrally ordered, capsid protein self-assembly is critical for Dengue virus replication as small molecules that target protein–protein interactions show antiviral activity [43,44].

Another means of breaking symmetry without large scale disorder is seen in the crystal structure of in vitro-assembled T = 1 Brome Mosaic Virus capsid. This structure was solved without icosahedral symmetry, although 60-fold non-crystallographic averaging was used [45]. When icosahedral symmetry was imposed, crystallographic statistics indicated a poor structure solution. The authors noted that protein–protein interactions in the T = 1 particle were sparse, suggesting that the particle was flexible leading to deformation of the particle by crystal packing. The naturally occurring T = 3 particle does appear to have icosahedral symmetry [46].

The biological asymmetry may be subtle. It may be intrinsic to the particle or induced during the viral lifecycle. For example, poliovirus RNA extrudes from a unique pentamer–pentamer interface, presumably due to the packing of the genome [47,48]. Parvoviruses bind transferrin receptor asymmetrically, suggesting either a unique feature on some subunits or that the capsid undergoes a conformational change upon receptor binding that results in asymmetry, i.e., an extreme negative cooperativity for binding more receptor [15].

**Figure 3:**
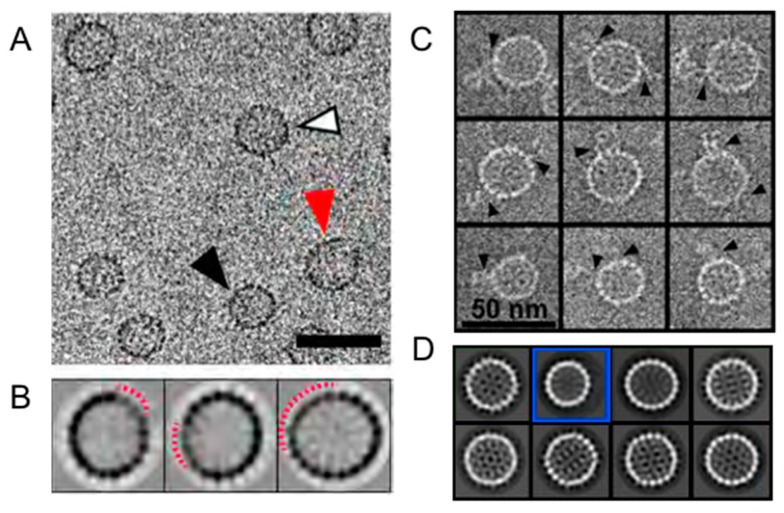
In vitro (**A**,**B**) assembly and (**C**,**D**) disassembly reactions with HBV capsids that exemplify defects in icosahedral symmetry. Without external information, one would not know that in both reactions T = 4 particles missing about 30 of the expected 120 dimers are prevalent. (**A**) Cryo-micrographs of HBV assembly reactions that were stalled due to conditions led to strong association energy and depletion of capsid protein. The micrograph shows normal T = 4 particles (white), disrupted T = 4 particles (red), and a T = 3 particle (black). The scale bar is 50 nm. (**B**) In reference free 2D class averages of T = 4 capsids from micrographs such as (**A**), the periphery of every class is disrupted, indicating that defects in particles dominate classification. The disrupted regions in the displayed classes are identified by the dashed red arcs. (**C**) Individual images from a negative stain electron micrograph of HBV capsids in a dissociation reaction induced by 1.2 M urea. Particle morphology was preserved by embedding in 0.1% trehalose. Many of these particles had “tails”, probably density from subunits falling off of capsids. (**D**) The first eight classes (in order of prevalence). Most classes are typical of T = 4 particles and display no obvious defects. Class 2 (blue box) is a T = 3 particle which make up about 10% of the total. Class 3 has an elliptical morphology not typically seen in HBV. Panels A and B are from Pierson et al. [20], and Panels C and D are from Lee et al. [53]. Figures are used with permission.

Single particle observations of capsid assembly and disassembly show that defects, irregular asymmetry, can arise and persist. During assembly of 120-dimer, T = 4 HBV capsids, it has been observed that incomplete particles of ≥90 dimers transiently accumulate and can be readily trapped by limiting the availability of free dimer (Figure 3) [20,49]. Surprisingly, in HBV and the closely related Woodchuck Hepatitis Virus assembly for a large fraction of particles is accompanied by overgrowth that may relax to a complete particle [50,51]. Because a large fraction of HBV particles formed in vivo are empty [52], the formation of empty capsids (even in vitro) is biologically relevant. A metastable species was also observed during HBV dissociation, as with assembly, also a ~90-dimer complex; however, no single type of holey capsid was observed in class averages of dissociation reactions, implying that they were an ensemble of structures [53]. A critical point is that these asymmetric particles, those that are overgrown and those that are holey, would have been obscured during reconstruction that removed non-ideal particles.

## 4. A New Perspective on Geometric Defects

Defects are everywhere. They should be put in perspective. We obtain our highest resolution structures by selecting the most regular particles and taking advantage of their symmetry, but ideal symmetry may not be needed or wanted for biological activity. We need to gain an understanding of how geometric defects can be characterized structurally and biologically.

From the perspective of structure determination: Asymmetric reconstruction and symmetric reconstruction each have advantages. Sixty-fold averaging provides an improvement of signal to noise that supports maximal resolution. Conversely, asymmetric reconstruction can identify unique features in an icosahedral background. With older equipment, i.e., a CCD (charge-coupled device) camera, asymmetric reconstructions have required substantial asymmetry, such as a phage tail [54] or MS2 phage bound to its receptor [18]; in the best cases, a polymerase was visualized for HBV [16]. If we look for them, defects have never been more experimentally accessible due to the combination of new hardware (direct electron detection cameras in particular [55,56]) that enhances high-resolution information and software that emphasizes maximum likelihood classification [5,57].

From a biological perspective: Identifying the selective advantage of defects will require formulation of new hypotheses. We have presented two in this perspective: (i) that defects in a symmetric capsid allow the virus to be more responsive to environment and (ii) that defects provide access to buried viral components, as in HIV. In both cases, defects may facilitate capsid structural transitions and uncoating. Defects effectively provide a labile “end” to a closed polymer, while still providing enough structural integrity to protect the viral genome. There is nuance between a particle with geometric defects and a defective particle. Defects may arise as scars from initiating or completing assembly; they may be incorporated stochastically during assembly; they may be imposed by interactions with virus- or host-derived molecules. Studies of the physics of defects in virus capsids, whether the defect leaves a large gap or a mismatch/disclinations, have shown that defects do not need to be irretrievably destabilizing [33], analogous to a liquid crystal. Nonetheless, it is not surprising that most of our examples of capsids with geometric defects come from enveloped viruses, where an envelope provides a compartment to protect a labile structure. The counter hypothesis, that geometric defects are serendipitous and rare mistakes in assembly, cannot be discounted in all cases but it should not be assumed. We speculate that mutations that favor or disfavor geometric defects will be isolated and their selective advantages can then be evaluated.
